# Imbalance between Glutamate and GABA in *Fmr1* Knockout Astrocytes Influences Neuronal Development

**DOI:** 10.3390/genes7080045

**Published:** 2016-08-10

**Authors:** Lu Wang, Yan Wang, Shimeng Zhou, Liukun Yang, Qixin Shi, Yujiao Li, Kun Zhang, Le Yang, Minggao Zhao, Qi Yang

**Affiliations:** 1 Department of Pharmacology, School of Pharmacy, Fourth Military Medical University, Xi’an 710032, China; 13324568099@163.com (L.W.); zsmapril_417@163.com (S.Z.); yujiao_li@126.com (Y.L.); kunzhang1900@163.com (K.Z.); yanglefmmu@163.com (L.Y.); 2 Department of Gastroenterology and Endoscopy Center, No. 323 Hospital of PLA, Xi’an 710054, China; vivian_27998@sina.com; 3 Fifth Company, Second Battalion, Cadet Brigade, Fourth Military Medical University, Xi’an 710032, China; liukun_yang@126.com (L.Y.); 1997sqx@sina.com (Q.S.)

**Keywords:** fragile X syndrome, FMRP, astrocyte, glutamate, oxidative stress, GABA

## Abstract

Fragile X syndrome (FXS) is a form of inherited mental retardation that results from the absence of the fragile X mental retardation protein (FMRP), the product of the *Fmr1* gene. Numerous studies have shown that FMRP expression in astrocytes is important in the development of FXS. Although astrocytes affect neuronal dendrite development in *Fmr1* knockout (KO) mice, the factors released by astrocytes are still unclear. We cultured wild type (WT) cortical neurons in astrocyte-conditioned medium (ACM) from WT or *Fmr1* KO mice. Immunocytochemistry and Western blotting were performed to detect the dendritic growth of both WT and KO neurons. We determined glutamate and γ-aminobutyric acid (GABA) levels using high-performance liquid chromatography (HPLC). The total neuronal dendritic length was reduced when cultured in the *Fmr1* KO ACM. This neurotoxicity was triggered by an imbalanced release of glutamate and GABA from *Fmr1* KO astrocytes. We found increased glutaminase and GABA transaminase (GABA-T) expression and decreased monoamine oxidase B expression in *Fmr1* KO astrocytes. The elevated levels of glutamate contributed to oxidative stress in the cultured neurons. Vigabatrin (VGB), a GABA-T inhibitor, reversed the changes caused by glutamate and GABA release in *Fmr1* KO astrocytes and the abnormal behaviors in *Fmr1* KO mice. Our results indicate that the imbalance in the astrocytic glutamate and GABA release may be involved in the neuropathology and the underlying symptoms of FXS, and provides a therapeutic target for treatment.

## 1. Introduction

Fragile X syndrome (FXS) is the most common form of inherited mental retardation, and is caused by a mutation in the fragile X mental retardation gene (*Fmr1*) [1]. The gene codes for the fragile X mental retardation protein (FMRP), which is critical for synaptic formation and plasticity [2,3,4,5]. FMRP is a mRNA binding protein that is associated with polyribosomes and involved in the translational efficiency and/or trafficking of certain mRNAs [6]. The *Fmr1* knockout (KO) mouse is a well-characterized model to study FXS-associated neuropathology and the underlying behavioral alterations [7,8,9].

To date, neuronal death has been given specific attention, with an increasing number of studies suggesting that the glial-neuronal interaction may be important in the development of FXS. There has been growing concern about the possible role of glia present in the brain [10]. Mounting evidence is steadily implicating a role that astrocytes play in neuronal dendritic development [11,12]. The role of astrocytes in the altered neurobiology of FXS was first demonstrated by Jacobs and Doering, who showed that *Fmr1* KO astrocytes have profound effects on dendrites such as reduced dendritic length and arbor area [13]. Our previous work showed that FMRP was expressed in the astrocyte lineage during development. The lack of FMRP in astrocytes leads to an overexpression of neurotrophin-3 (NT-3), which, in turn, induces the deficit of dendritic growth in neurons [14]. However, the role of neurotoxic molecules released from *Fmr1* KO astrocytes following neuronal damage remains unclear.

Studies report that drugs used to treat excessive glutamate and insufficient γ-aminobutyric acid (GABA), signaling pathways affected by FMRP, are under different stages of development in FXS [15]. Thus, it is interesting to know whether the imbalance between excitatory and inhibitory circuitry is caused by a lack of astrocytic FMRP. Glutamate is the most abundant excitatory neurotransmitter in the brain. Increased levels of extracellular glutamate cause over-stimulation of glutamate receptors that may result in secondary events, leading to neuronal cell death or “excitotoxicity” [16]. Excessive glutamate levels have also been directly attributed to oxidative stress [17,18,19], which has been linked to the neuropathology of neurodegenerative disorders, stroke, trauma, seizures, and age-related cognitive deficits [20,21].

In the present study, we report that increased glutamate and reduced GABA levels were neurotoxic to neuronal dendritic development. The neurotoxicity resulted from an imbalance between the glutamate and GABA circuits in the brain, which contributes to oxidative stress in FXS. The imbalanced condition is partly due to the increased glutaminase and decreased Monoamine Oxidase B (MAOB) in *Fmr1* KO astrocytes, particularly with elevated levels of GABA transaminase (GABA-T). Treatment with vigabatrin (VGB) can restore the imbalance through irreversible inhibition of GABA-T, and reverse the deficits in locomotor activity and trace fear memory in *Fmr1* KO mice. Our findings in this study suggest a new potential strategy for FXS treatment.

## 2. Materials and Methods 

### 2.1. Animals

*Fmr1* WT and KO mice of the FVB.129P2-Fmr1tm1Cgr strain were maintained as described previously [14,22]. All animal protocols used were approved by the Animal Care and Use Committee of the Fourth Military Medical University.

### 2.2. Primary Neural Cultures

Primary astrocytes were isolated from newborn (1–2 days old) *Fmr1* WT or KO mice using the differential adhesion method [14,23]. In our preparations, over 95% of the adherent cells were astrocytes as demonstrated by anti-glial fibrillary acidic protein (GFAP) immunostaining. To obtain astrocyte-conditioned medium (ACM), astrocytes were first cultured in 24-well culture plates for 24 h in Dulbecco’s modified Eagle medium (DMEM) with 10% fetal bovine serum. Cultures were washed extensively with Hank’s balanced salt solution (HBSS; Invitrogen, Carlsbad, CA, USA) and changed to neurobasal medium with B27 supplementation (NB/B27; Invitrogen, Carlsbad, CA, USA) by adding 500 μM glutamine. The NB/B27-based medium was conditioned for 7 days and collected after brief centrifugation. During this period, the medium had few effects on astrocyte proliferation and cell viability [24]. Prior to exposure on neuron culture, the ACM had an osmolarity of 292 mOsm/kg and pH of 6.80.

The cortical neurons were prepared from 15-day-old embryos (E15) of WT mice according to a previously described method [14,25]. Neurons were cultured in NB/B27 with half of the medium changed every 3 days. For ACM culturing, neurons were plated for 4 h and then replaced by a 1:1 mixture of ACM and neuronal maintenance medium.

### 2.3. Drug Treatment

VGB (Tocris Bioscience, Missouri, USA), a GABA-T inhibitor, was dissolved in saline (vehicle), and administered daily to *Fmr1* KO astrocytes that were cultured in NB/B27 at a dose of 100 μM. The ACM was obtained after 7 days and collected after brief centrifugation to measure glutamate and GABA levels. The cells were used for GABA-T determination. Male mice (3–4 weeks old) received an oral administration of vehicle or VGB (25 mg/kg, p.o.) twice a day for 6 days. Behavioral tests were performed during the experiments.

### 2.4. Immunocytochemistry

Cells were fixed in 4% paraformaldehyde and stained with rabbit anti-MAP2 (Anti-Microtubule-Associated Protein 2) (1:1000, Millipore Bioscience Research Reagents, Bellerica, MA, USA) overnight at 4 °C, followed by incubation with secondary antibodies conjugated with Cy3 (1:200, Boster Bio-Technology, Wuhan, China). Images were collected using an Olympus confocal laser scanning FV1000 microscope (Olympus, Tokyo, Japan). Dendritic complexity was assessed by Sholl analysis [26]. Here, a series of concentric rings of consistently increasing size are centered over the cell soma and the number of dendritic crossings is measured at each ring. Dendritic arbors with more branches will have an increased number of ring crossings, thus providing a quantitative assessment of dendritic complexity [27]. The photography and analysis of immunoreactivity with ImageJ software (National Institutes of Health) were carried out in an investigator-blinded manner for the three independent experiments.

### 2.5. Western Blot Analysis

Cells were washed with ice-cold PBS and incubated with RIPA buffer (Beyotime Institute Biotechnology, Shanghai, China) containing a proteinase inhibitor mixture (Roche, Mannheim, Germany) and 10 μM PMSF (Sigma, St. Louis, MO, USA). Lysates were briefly sonicated and cleared by centrifugation at 10,000 rpm for 20 min. Equivalent amounts of protein were analyzed using 10% SDS-PAGE gel electrophoresis. Proteins were transferred to polyvinylidene difluoride membranes and probed with antibodies. The following primary antibodies were used: anti-MAP2 (1:1000; Millipore Bioscience Research Reagents, Billerica, MA, USA), anti-PSD95 (postsynaptic density protein 95) (1:2000; Abcam, Cambridge, UK), anti-GluR1 (Glutamate Receptor 1) (1:300; Abcam), anti-MAOB (1:1000; Abcam), anti-GFAP (1:1000; Abcam), anti-GABA-T (1:1000; Abcam), anti-glutaminase (1:1000, Abcam), anti-β-actin (1:10000, Sigma). Secondary antibodies were HRP-conjugated anti-rabbit or anti-mouse antibodies (1:10000, Boster Bio-Technology, Wuhan, China). Visualization was performed using enhanced chemiluminescence (ECL, GE Healthcare Pharmacia). Densitometric analysis of Western blots was conducted using a ChemiDoc XRS (Bio-Rad, Hercules, CA, USA) and quantified using Quantity One version 4.1.0 (Bio-Rad).

### 2.6. Measurement of Glutamate and GABA

The level of glutamate and GABA in the ACM was analyzed by high-performance liquid chromatography (HPLC) using a Waters 2695 liquid chromatograph (Waters, Milford, CT, USA), a Hypersil ODS C18 column (250 by 4.6 mm; particle size, 5 μm), and a Waters 2996 UV/VIS detector. Before injection into the HPLC, the sample was derivatized by incubation with 2,4-dinitrofluorobenzene for 1 h at 60 °C and the reaction was stopped by adding 50 mM potassium dihydrogen phosphate. The mixture was concentrated and filtered using a 0.22 μm filter. The mobile phase consisted of 8% methyl cyanides, 8% ddH_2_O, and 84% 40 mM sodium acetate (pH 8.0) at a flow rate of 1.0 mL/min. The UV detector was set to an absorbance of 360 nm. Chromatograms of glutamate and GABA standards yielded respective peaks at 7.968 min and 29.896 min. The concentrations were calculated by LCsolution software (Waters, Milford, CT, USA) based on standard samples.

### 2.7. Determination of Thiobarbituric Acid-Reactive Substances

The measurement of malondialdehyde (MDA), the most abundant product arising from lipid peroxidation, has been extensively used as an index of oxidative stress [28,29]. The amount of thiobarbituric acid-reactive substances (TBARS), was estimated by spectrophotometric absorbance at 532 nm (DU-800, Beckman Coulter, Fullerton, CA, USA). The absorbance measurements were calculated using a standard curve using 10 nmol/mL MDA (Jiancheng Bioengineering Institute, Nanjing, China).

### 2.8. Measurement of Total Reactive Oxygen Species (ROS) Production

The production of intracellular ROS was analyzed by quantifying 2′,7′-dichlorofluorescein diacetate (DCFH-DA; Beyotime Institute Biotechnology, Shanghai, China) [30,31]. Briefly, after being cultured with ACM for 7 days, neurons were incubated with 10 μM DCFH-DA at 37 °C in the dark for 20 min. The cells were then rinsed twice with HBSS, and the fluorescence intensity was detected using a spectrofluorometer (F-2500, Hitachi, Tokyo, Japan) at an excitation wave length of 488 nm and an emission wave length of 535 nm.

### 2.9. Open Field Test

The open field (JLBehv-LAM-4, Jiliang, Shanghai, China) was a square arena (30 × 30 × 30 cm) with clear Plexiglas walls and floor. It was placed inside a dimly illuminated isolation chamber with a fan. Before the experiment, mice were habituated to experimental conditions, but not to the open field. Prior to formal testing, mice were placed in the center of the box and allowed to freely explore for 2 min to adjust to the open field. For testing, each animal was placed in the center of the open field and measured for 15 min. The exploratory behaviors of mice were videotaped using a camera fixed above the floor and analyzed with a video-tracking system.

### 2.10. Trace Fear Memory

Trace fear conditioning was performed in an isolated shock chamber (Med Associates, St. Albans, VT, USA). The conditioned stimulus (CS) used was an 80 dB white noise, delivered for 15 s, and the unconditioned stimulus (US) was a 0.7 mA scrambled footshock for 0.5 s. Mice were acclimated for 60 s and were presented with 10 CS–trace–US intertrial interval (ITI) trials (trace, 30 s; ITI, 210 s). One day after training, mice were acclimated for 60 s followed by 10 CS–ITI trials (ITI, 210 s) in a novel chamber to test for trace fear memory. All data were video recorded using FreezeFrame Video-Based Conditioned Fear System and analyzed by Actimetrics Software (Coulbourn Instruments, Wilmette, IL, USA). Average freezing for the baseline and for each ITI during the training and testing sessions was analyzed. Bouts of 1.0 s were used to define freezing.

### 2.11. Statistical Analyses

Statistical analyses were conducted using SigmaPlot 2001 (Systat software, Point Richmond, CA, USA), with an α level of 0.05. Data are presented as mean values ± SEM. Analysis of variance (ANOVA) was used to compare quantitative values from cultures across groups. Tukey’s studentized range test was used to adjust for multiple comparisons in post hoc pairwise tests.

## 3. Results

### 3.1. Fmr1 KO ACM Is Not Conducive to Dendritic Development

**Figure 1:**
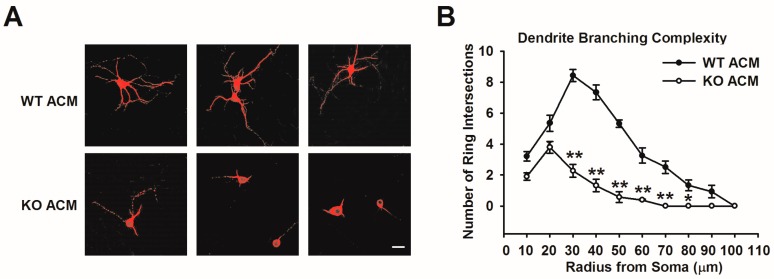
Altered dendrite branching complexity of neurons cultured with *Fmr1* KO ACM. (**A**) Representative images of dendritic arborization in individual neurons cultured with WT (upper) and KO (lower) ACM. Scale bar = 50 μm; (**B**) Branching complexity of apical and basilar dendrites were quantified using Sholl analysis as the number of ring intersections by dendritic branches with the ring diameter set at 10 μm. The overall pattern of branching was less complex for KO ACM-treated neurons. The number of neurons for WT ACM and KO ACM was 45 and 48, respectively. Data are from three independent experiments. * *p* < 0.05, ** *p* < 0.01 compared with WT ACM.

*Fmr1* KO mice have abnormal dendrite and spine morphology in the neocortex and hippocampal dentate gyrus [32,33]. These changes may underlie neurologic features found in individuals with fragile X-associated tremor/ataxia syndrome. Our previous study demonstrated that KO astrocytes induce abnormal dendritic development through the secretion of soluble factors in ACM [14]. KO ACM-cultured neurons show reduced total dendritic length [14]. To eliminate the possibility that neuronal density may underlie the aberrant neuronal morphology caused by KO ACM, we prepared low-density cultures to visualize individual neurons using staining with the dendritic marker, MAP2 (Figure 1A). Dendritic complexity was quantified with Sholl analysis to count the number of branch intersections with concentric rings progressively increasing in diameter by 10 μm. As shown in Figure 1B, the number of ring intersections was generally lower in KO ACM-cultured neurons compared with WT ACM. The changes at a single cell level indicate that *Fmr1* KO ACM alters the neuronal morphology.

### 3.2. Fmr1 KO Astrocytes Result in an Imbalanced Release of Glutamate and GABA

**Figure 2:**
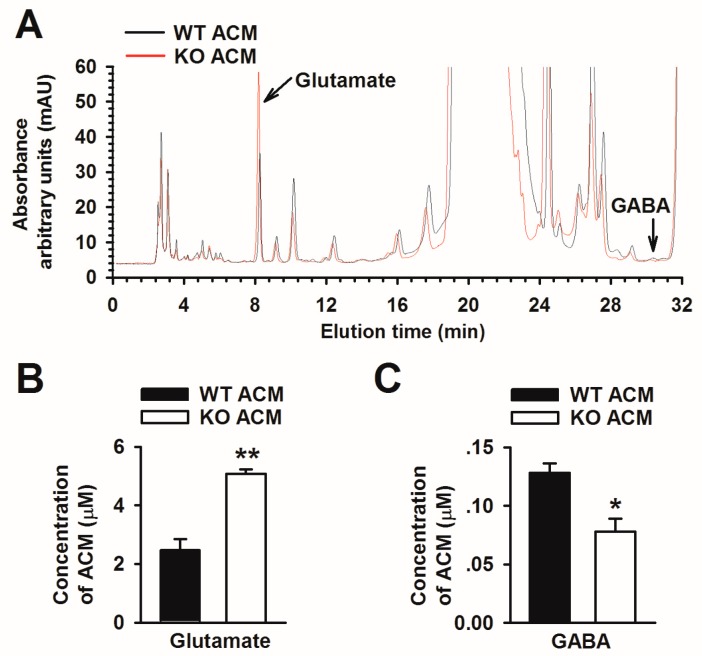
*Fmr1* KO astrocytes release an unusual level of glutamate and GABA. (**A**) HPLC chromatograms of glutamate and GABA from WT and KO ACM. Chromatogram in red represents KO ACM and the black represents WT ACM. The absorbance was measured in arbitrary units (mAU). All elution profiles contained two peaks that eluted at 7.968 min and 29.896 min, which were the components of glutamate and GABA, respectively; (**B**) The level of glutamate was significantly increased in KO ACM. Data are from three independent experiments; (**C**) The level of GABA was significantly decreased in KO ACM. Data are from three independent experiments. * *p* < 0.05, ** *p* < 0.01 compared with WT ACM.

Studies report that astrocyte-derived glutamate could act on extrasynaptic *N*-methyl-d-aspartic acid (NMDA) receptors, causing excitotoxic neuronal damage [34,35,36]. We used HPLC to analyze glutamate and GABA levels in WT and KO ACM. HPLC analysis revealed that glutamate and GABA were eluted at 7.968 min and 29.896 min, although the GABA peak in the chromatogram was not readily visible (Figure 2A). The levels of glutamate and GABA in ACMs were calculated referring to the standard curve *y* = 3212.2*x* − 6025.9 (R^2^ = 0.9999) and *y* = 2056.4*x* + 5230.3 (R^2^ = 0.9997), respectively. The level of glutamate in KO ACM was 5.08 ± 0.15 μM, significantly higher than that in WT ACM (2.48 ± 0.36 μM) (Figure 2B). However, the GABA concentration in KO ACM was 0.08 ± 0.01 μM, significantly lower than that in WT ACM (0.13 ± 0.02 μM) (Figure 2C). This result suggests that *Fmr1* KO astrocytes disturb the balance of excitatory/inhibitory transmitter release.

### 3.3. Excessive Glutamate Results in Abnormal Neuronal Growth

**Figure 3:**
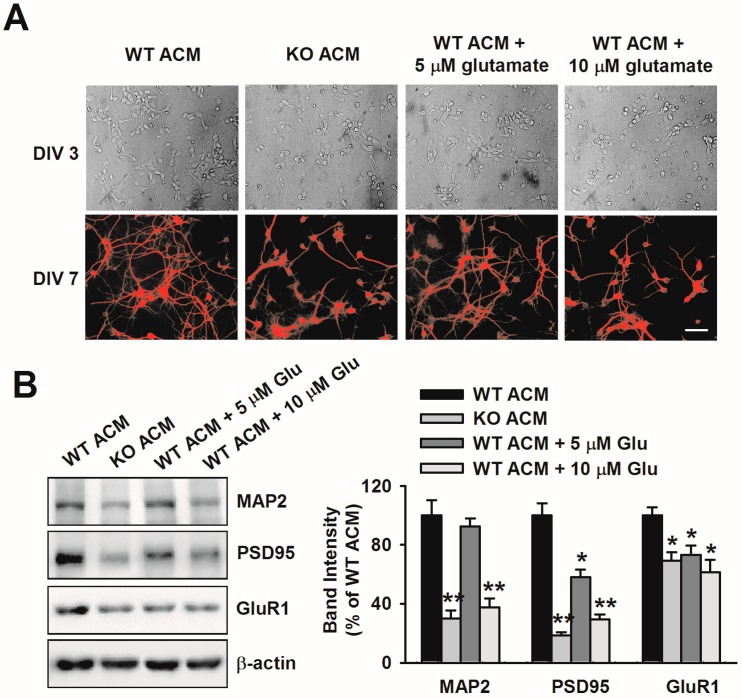
Excessive glutamate in *Fmr1* KO ACM mediated the abnormal neuronal development. (**A**) Representative phase contrast and immunofluorescent images in which the dendrites were demonstrated by immunostaining for MAP2. At either DIV 3 or 7, glutamate-treated neurons showed stunted dendritic morphology, similar to KO ACM-treated neurons. Scale bar = 50 μm; (**B**) Western blot analysis of MAP2, PSD95, and GluR1 in ACM-cultured neurons after glutamate treatment. Band intensities were quantified as a percentage of values from WT ACM-treated neurons. *n* = 6 wells from three independent experiments. * *p* < 0.05, ** *p* < 0.01 compared with WT ACM.

To determine whether glutamate contributes to the dendritic disorder caused by KO ACM, we added exogenous glutamate into WT ACM and examined the neuronal morphology. At Days in Vitro (DIV) 3, neurons treated with 5 μM and 10 μM glutamate showed robust signs of dendritic damage including shorter dendrites and stunted arborization, which was similar to KO ACM cultured neurons (Figure 3A). At DIV 7, immunofluorescent staining for MAP2 showed a dramatic reduction of the dendrite lengths (Figure 3A). However, cell viability was unchanged by the addition of glutamate. In addition to the disrupted morphology, glutamate or KO ACM significantly decreased the levels of postsynaptic elements of excitatory synapses, specifically MAP2, PSD95, and GluR1 (Figure 3B). Taken together, these data indicate that excessive glutamate can cause damage to dendritic development.

### 3.4. Excessive Glutamate Contributes to Oxidative Stress

**Figure 4:**
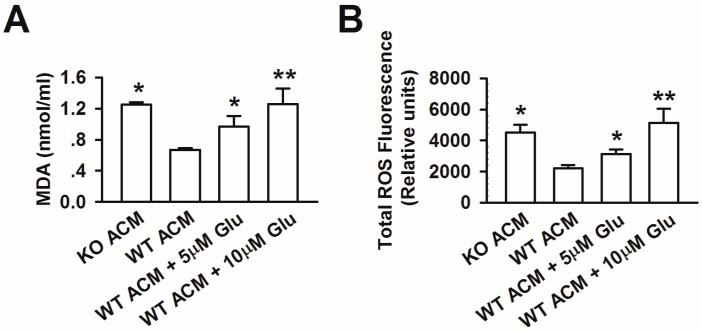
Oxidative stress was involved in neuronal excitotoxicity triggered by glutamate. (**A**) MDA levels in culture mediums were analyzed using a spectrophotometer. Glutamate increased the MDA levels in a dose-dependent manner. Data are from three independent experiments. * *p* < 0.05, ** *p* < 0.01 compared with WT ACM; (**B**) The total ROS production was measured using a spectrofluorometer. Glutamate increased the ROS production in a dose-dependent manner. Data are from three independent experiments. * *p* < 0.05, ** *p* < 0.01 compared with WT ACM.

We further explored the possible consequences of glutamate-induced neurotoxicity. We hypothesized that high levels of glutamate contribute to the oxidative stress in FXS, as supported by other studies [21,37]. In order to characterize the oxidative damage caused by glutamate, we measured MDA and intracellular ROS in neurons cultured with ACM for 7 days. We found that the levels of MDA and ROS were significantly increased in WT ACM-cultured neurons after glutamate treatment, which is similar to KO ACM-cultured neurons (Figure 4A,B). In addition, the production of MDA and ROS correlated with the dose of glutamate, which suggested moderate oxidative stress. These results indicate that the elevation of glutamate may be one of the factors inducing oxidative damage in FXS.

### 3.5. Altered Glutamate and GABA Metabolic Enzymes in Fmr1 KO Astrocytes

**Figure 5:**
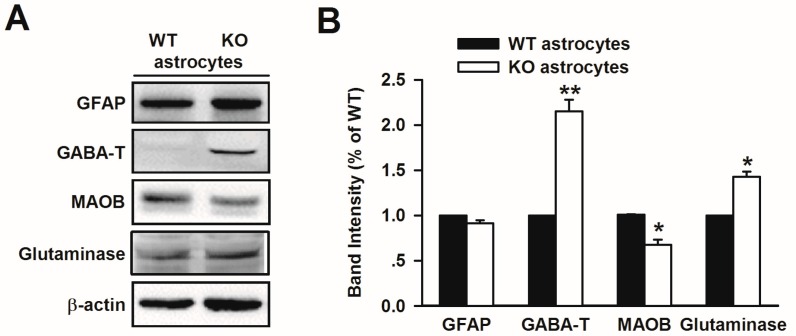
Increased GABA-T and glutaminase, and reduced MAOB expression in *Fmr1* KO astrocytes. (**A**) Western blot analysis of GABA-T, glutaminase, and MAOB from cultured astrocytes of WT and KO mice; (**B**) Band intensities were quantified as a percentage of values from WT astrocytes. *n* = 6 wells from three independent experiments. * *p* < 0.05, ** *p* < 0.01 compared with the WT astrocytes.

To investigate the mechanism underlying elevated glutamate and reduced GABA release by *Fmr1* KO astrocytes, we focused on glutaminase, which produces glutamate from glutamine and is a major enzyme for glutamate synthesis in the glia and in the GABA metabolizing enzyme, GABA-T [38,39]. Furthermore, the MAOB-mediated putrescine degradation pathway is responsible for GABA production in reactive astrocytes [10]. Therefore, we detected the expression of the enzymes in WT and KO astrocytes. Western blotting showed that levels of glutaminase and GABA-T in KO astrocytes were higher than the levels in WT astrocytes. Meanwhile, the protein level of MAOB in KO astrocytes was lower than the level in WT astrocytes (Figure 5). Collectively, our results suggest that glutaminase, GABA-T, and MAOB are involved in the imbalanced release of glutamate and GABA by *Fmr1* KO astrocytes.

### 3.6. Treatment with VGB Rescues the Abnormal Behaviors of Fmr1 KO Mice

**Figure 6:**
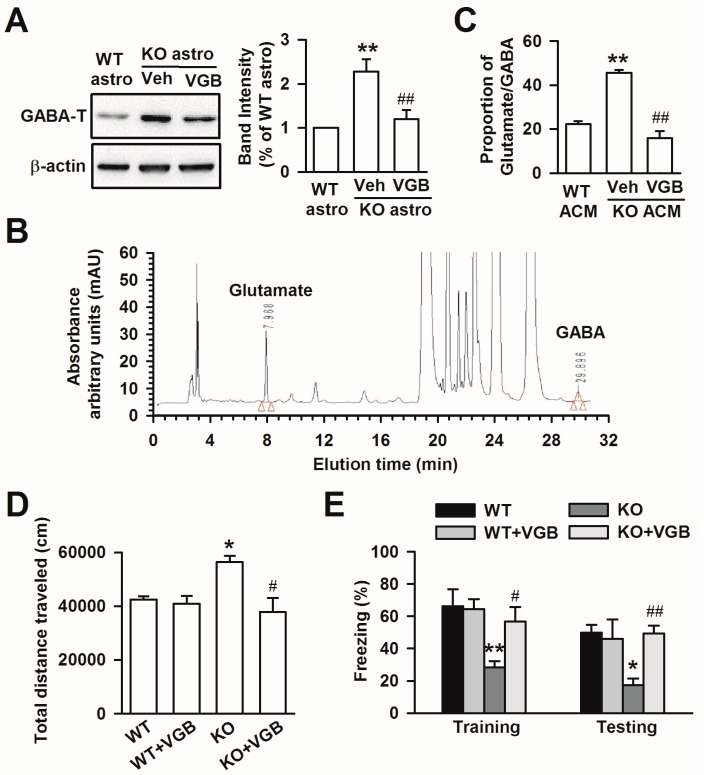
Treatment with VGB inhibited GABA-T expression and reversing the abnormal behaviors caused by *Fmr1* KO astrocytes. (**A**) Band image and intensity analysis of GABA-T from cultured astrocytes showed that VGB inhibited GABA-T expression in KO astrocytes. *n* = 6 wells from three independent experiments. ** *p* < 0.01 compared with the WT astrocytes, ## *p* < 0.01 compared with the vehicle-treated KO astrocytes; (**B**) HPLC chromatograms of glutamate and GABA after VGB treatment in KO ACM; (**C**) The proportion of glutamate/GABA was significantly decreased after VGB treatment in KO ACM. Data are from three independent experiments. ** *p* < 0.01 compared with WT ACM, ## *p* < 0.01 compared with KO ACM + vehicle group; (**D**) Open-field test demonstrating the total traveled distance by the mice (*n* = 8 mice in each group). * *p* < 0.05 vs. WT mice, # *p* < 0.05 vs. KO mice; (**E**) Fear conditioning task (*n* = 8 mice in each group). Oral administration of VGB (25 mg/kg, twice a day for 6 days) to KO mice significantly increased freezing time both during training and testing. * *p* < 0.05, ** *p* < 0.01 vs. WT mice, # *p* < 0.05, ## *p* < 0.01 vs. KO mice.

VGB is an antiepileptic drug that acts by inhibiting the catabolism of GABA to glutamate. It is approved to treat refractory complex partial seizures [40]. VGB (100 μM) significantly reduced the GABA-T level in *Fmr1* KO astrocytes (Figure 6A). HPLC showed that VGB treatment reversed the altered glutamate and GABA release in KO ACM (Figure 6B). After VGB treatment, the glutamate to GABA ratio in KO ACM decreased to 5.86 ± 3.15, which is similar to that seen in WT ACM (22.30 ± 1.37) (Figure 6C). Furthermore, oral administration of 25 mg/kg VGB twice a day for six consecutive days decreased the locomotor activity of *Fmr1* KO mice (Figure 6D). In trace fear condition, *Fmr1* KO mice displayed slightly increased freezing throughout the training session. After VGB administration, *Fmr1* KO mice successfully learned trace fear conditioning after 10 CS–US pairings and showed similar freezing when compared with WT mice (Figure 6E). These results suggest that GABA-T may be a potential therapeutic target for FXS.

## 4. Discussion

Fragile X syndrome is caused by the loss of FMRP due to the expansion of a CGG repeat in the promoter region of the *Fmr1* gene [41]. Because of the predominant behavioral and cognitive abnormalities associated with it, FXS is generally considered to be a neuronal disorder. However, emerging evidence strongly suggests that FMRP is also expressed in astrocytes, oligodendrocytes, and microglia, in addition to neurons [11,42]. Our previous study has shown the importance of astrocytes in neuronal development by regulating the secretion of NT-3 [14]. The current study focused on neurotoxic factors released by *Fmr1* KO astrocytes. We found that the neurotoxicity was triggered by excessive glutamate and reduced GABA levels, as a consequence of enhanced glutaminase and GABA-T expression in astrocytes and decreased MAOB level, which contributed to the oxidative stress in FXS.

A number of studies have demonstrated that glia support neighboring neurons in various ways. For example, astrocytes secrete thrombospondins to promote synapse formation in CNS synaptogenesis [43], regulate the stability and maturation of dendritic spines [44,45], and take part in the regulation of synaptic plasticity and synaptic transmission [46,47]. Thus, astrocytes are involved in many crucial functions of the nervous system and are associated with several neurodegenerative diseases, including amyotrophic lateral sclerosis, Rett syndrome, Huntington’s disease, and Parkinson’s disease. In this study, we demonstrated that *Fmr1* KO astrocytes detrimentally influence neuronal dendrite formation in a non-cell-autonomous manner. The issue of whether KO astrocytes were over-activated was not addressed in our study, although another study has reported increased expression of the astrocyte marker GFAP in the cerebellum of *Fmr1* KO mice [48]. However, we showed that using immune blotting, the expression of GFAP was similar between cultured WT and KO astrocytes. This may be because of the absence of immune stressors in vitro.

Astrocytes modulate synaptic neurotransmission by releasing glutamate and GABA. Excessive glutamate leads to neurotoxicity [49,50]. We identified glutamate as the toxic factor released from *Fmr1* KO astrocytes based on the following evidence: (1) KO astrocytes released more glutamate; (2) the addition of excess glutamate to WT ACM induced an abnormal neuronal morphology similar to the KO ACM. It is known that a higher rate of production and release of glutamate or a lower rate of its uptake in the glia, can lead to a high extracellular level of glutamate [38]. To investigate the hypothesis of a higher rate of glutamate production, we focused on glutaminase, the principal enzyme responsible for the production of glutamate from glutamine in astrocytes. Previous reports showed that glutaminase is responsible for generating excess glutamate, causing toxicity in cultured neurons [51,52]. We found that the expression of glutaminase was higher in cultured KO astrocytes than in WT, which was consistent with the in vivo data. Another participant, GABA-T, was also overexpressed in KO astrocytes. VGB, which inhibits the catabolism of GABA by irreversibly inhibiting GABA-T [40], reversed the high glutamate release in KO astrocytes. Furthermore, we found that the expression of MAOB, a main enzyme for GABA production, was reduced in KO astrocytes. This reduction induced insufficient GABA production and aggravated the imbalance of glutamate and GABA. Thus, our findings suggest the connected mechanism between excess glutamate and low GABA released by *Fmr1* KO astrocytes. However, other mechanisms may be potentially involved, such as exocytosis or glutamate transporter-mediated neurotransmitter reuptake by astrocytes, and remain to be studied [53,54,55].

Oxidative stress plays a critical role in mediating glutamate-induced neurotoxicity [56]. ROS remarkably decreases the number of dendrites, but does not induce cell death [57]. In particular, oxidative stress has been implicated in a number of neurodegenerative pathologies, including FXS, which results in altered protein conformation and function [21,58]. Previous studies in *Fmr1* KO mice have suggested increased ROS generation, NADPH-oxidase activation, and oxidative stress [21,29]. In this study, oxidative stress was increased in FXS. The levels of TBARS and ROS were increased in the WT ACM after glutamate treatment.

The present data demonstrate that the aberrant release and uptake of neurotransmitters by *Fmr1* KO astrocytes influences neuronal dendritic development. However, the function of FMRP in astrocytic neurotransmitter production is not well understood. We hypothesize that FMRP, as a RNA binding protein, negatively regulates the expression of glutamate and GABA-related molecules in astrocytes. The excitotoxic mechanism of FXS pathology detailed in this study suggests that GABA-T inhibitors may be of clinical value; however, further research is required to clarify this issue. Finally, the present study provides a possible explanation for the role of glia in the abnormal neurodevelopment of FXS and represents a new experimental approach for its treatment.
